# Episodic Tags Enhance Striatal Valuation Signals during Temporal Discounting in pathological Gamblers

**DOI:** 10.1523/ENEURO.0159-17.2017

**Published:** 2017-06-13

**Authors:** Antonius Wiehler, Frederike Hermi Petzschner, Klaas Enno Stephan, Jan Peters

**Affiliations:** 1 Department of Systems Neuroscience, University Medical Center Hamburg-Eppendorf, 20246 Hamburg, Germany; 2 Translational Neuromodeling Unit, Institute for Biomedical Engineering, University of Zurich and ETH Zurich, 8032 Zurich, Switzerland; 3 Wellcome Trust Centre for Neuroimaging, University College London, London, WC1N 3BG United Kingdom; 4 Max Planck Institute for Metabolism Research, 50931 Cologne, Germany; 5 Department of Psychology, Biological Psychology, University of Cologne, 50923 Cologne, Germany

**Keywords:** addiction, decision making, episodic future thinking, fMRI, pathological gambling, temporal discounting

## Abstract

Similar to many addiction disorders, pathological gambling is associated with an increased preference for immediate rewards (steep temporal discounting). In healthy participants, episodic future thinking has been shown to reduce impulsivity during intertemporal choice. Here, we examine for the first time a modulation of temporal discounting via episodic future thinking in a group of pathological gamblers. We investigated a sample of 24 pathological gamblers and 24 matched healthy controls with functional magnetic resonance imaging (fMRI). Participants made intertemporal choices in two experimental conditions. In the control condition, delayed monetary rewards were offered with the respective amount and delay. In the episodic condition, rewards were additionally associated with participant-specific personal future events. We replicated previous findings of increased temporal discounting in pathological gambling. On a trend level, episodic future thinking attenuated discounting across all participants. We found that pathological gamblers could successfully recruit a prospection related network during decision-making in the presence of episodic information. The episodic condition modulated the functional connection between ventromedial prefrontal cortex (vmPFC) and ventral striatum, a mechanism that might support the increase in striatal value coding observed in the episodic condition in gamblers. However, in controls, but not in gamblers, valuation signal changes in the hippocampus were associated with less impulsive behavior. We provide first evidence that by episodic cues during intertemporal decision-making striatal valuation signals can be enhanced in pathological gamblers. Further research is needed to explore interventions that reliably reduce impulsive choice behavior in pathological gambling.

## Significance Statement

Psychiatry research has observed steep temporal discounting in many forms of addiction, including pathological gambling. At the same time, the nature of valuation signals in the ventral striatum in pathological gambling is debated. In healthy participants, episodic future thinking modulates temporal discounting. Baseline episodic future thinking has been found to be unimpaired in pathological gamblers. This raises the possibility for similar modulation effects in this clinical group. We found no evidence for an impairment of episodic future thinking and related brain activity in pathological gamblers. By triggering episodic future thinking during temporal discounting, we demonstrate for the first time an experimental paradigm that enhances striatal valuation signals in pathological gamblers in a nongambling context.

## Introduction

An increased preference for immediate rewards is a hallmark feature of addiction and has been observed in opioid (Madden et al., 1997; Kirby et al., 1999), cocaine (Coffey et al., 2003), alcohol (Myerson et al., 2015), nicotine (Bickel et al., 1999), and gambling (Bickel et al., 2014) addiction. Such reward devaluation by time has been studied extensively in the framework of temporal discounting (Green and Myerson, 2004; Bickel et al., 2014). pathological gambling, a disorder recently defined as a behavioral addiction (American Psychiatric Association, 2013), is similarly associated with increased temporal discounting (Petry, 2001; Madden et al., 2011; Miedl et al., 2012; Wiehler and Peters, 2015).

Neuronal deficits have been identified as contributors to steep temporal discounting in pathological gambling. Agents choose between smaller-but-sooner (SS) and larger-but-later (LL) rewards by comparing subjective (discounted) reward values. These values are thought to be computed via a subjective integration process of the option dimensions (in this case amount and delay) in nucleus accumbens (NAcc), posterior cingulate cortex (PCC) and ventromedial prefrontal cortex (vmPFC; Bartra et al., 2013; Clithero and Rangel, 2014). Projections from vmPFC to NAcc exist in both primates and humans (Haber and Knutson, 2010). However, whether neuronal valuation processes are decreased or increased in pathological gambling is a matter of ongoing debate with inconsistent findings (Hewig et al., 2010; Balodis et al., 2012a; Van Holst et al., 2012; Clark et al., 2013). The heterogeneity between studies might be due to the prominent modulator role of contextual factors in addiction (Leyton and Vezina, 2013). An impaired valuation of LL rewards might contribute to steep discounting in pathological gamblers (Miedl et al., 2012).

It is an open question whether episodic future thinking can modulate temporal discounting in pathological gamblers. Episodic future thinking (also referred to as prospection; Gilbert and Wilson, 2007) is the ability to project oneself into the future and to imagine possible future episodes (Addis et al., 2009). Although possibly not a requirement for self-control (Kwan et al., 2012), it has been proposed that episodic future thinking can modulate decision-making via hippocampal involvement in simulating possible future outcomes (Schacter et al., 2007; Bar, 2009). When episodic future thinking is cued with personal future events during temporal discounting, healthy participants discount rewards less steeply than in a control condition without cues, while showing an increased prefrontal-hippocampal coupling (Peters and Büchel, 2010; Benoit et al., 2011). In line with this view, medial temporal lobe lesions impair episodic future thinking (Hassabis et al., 2007; Race et al., 2011) and attenuate interactions of episodic future thinking with temporal discounting (Palombo et al., 2015; but see Kwan et al., 2015), whereas baseline discounting is unaffected (Kwan et al., 2012; Palombo et al., 2015).

However, knowledge is limited in addiction. First, an intact baseline episodic future thinking is a requirement for these putative interactions between episodic future thinking and temporal discounting. While opioid addicts might be impaired in episodic future thinking (Mercuri et al., 2015), episodic future thinking is unaffected and unrelated to baseline temporal discounting in pathological gambling (Wiehler et al., 2015). In this previous study, patients imagined possible future events following a cue. The number of episodic details in their narration was analyzed as a proxy for episodic future thinking (Levine et al., 2002; Race et al., 2011). Second, regarding an interaction of episodic future thinking and temporal discounting, a study in alcohol dependent patients showed an attenuating effect of episodic future thinking on temporal discounting behavior (Snider et al., 2016).

Given the widely observed attenuating effect of episodic future thinking on temporal discounting in healthy controls and the unimpaired episodic future thinking in pathological gamblers, an important open question is whether temporal discounting can be attenuated via episodic future thinking in these patients. As baseline episodic future thinking was previously shown to be unimpaired in pathological gamblers, we expected to find consistent activation (i.e., overlapping networks) in pathological gamblers and healthy controls during episodic future thinking, a hypothesis we tested with a conjunction analysis. We further hypothesized that interactions between episodic future thinking and temporal discounting would be reduced in pathological gamblers, which, in turn, we expected to contribute to steeper temporal discounting in pathological gambling. Accordingly, we expected diminished brain valuation signals in pathological gambling.

## Materials and Methods

### Participants

We investigated a final sample of *n* = 24 nontreatment-seeking pathological gamblers fulfilling the DSM-5 criteria and *n* = 24 healthy controls (all male) with functional magnetic resonance imaging (fMRI). Groups were matched on age, income, education and nicotine use. An additional *n* = 7 pathological gamblers and *n* = 8 controls completed the same task without fMRI scanning in a quiet behavioral lab on a PC (“behavioral pilots”). All participants were recruited via adverts posted on local Internet bulletin boards. One pathological gambler was excluded due to comorbid Axis I disorders and one healthy control participant was excluded due to unusually high brain atrophy of unknown cause.

All participants reported no history of psychotropic drugs nor regular drug use except for nicotine. Current drug abstinence was verified via urine drug screening. No axis I disorder was present in the sample, except for depression (seven pathological gamblers, three healthy controls). All pathological gamblers met the DSM-5 criteria of pathological gambling and met the criteria in the Kurzfragebogen zum Glücksspielverhalten (KFG; Petry, 1996) and the German version of the South Oaks gambling screen (SOGS; Lesieur and Blume, 1987). Eleven pathological gamblers and 10 healthy controls were current smokers (>4 in the Fagerström test for nicotine dependence, FTND; Heatherton et al., 1991) and were allowed to smoke freely before testing (see Table 1 for sample characteristics).

**Table 1.** Overview about sample characteristics

	Pathological gamblers		Healthy controls		Group comparison		
	Mean	SD	Mean	SD	*t*	df	*p*
Age	29.68	10.88	28.47	7.13	0.52	51.51	0.61
School years	11.16	1.55	11.25	1.50	-0.23	60.75	0.82
Monthly income	1282.87	668.10	1084.50	608.90	1.23	60.07	0.22
FTND	3.13	2.47	3.66	2.10	-0.91	58.83	0.37
AUDIT	9.06	6.55	6.69	3.94	1.74	48.88	0.09
DSM-5 score	5.81	1.45	0.22	0.55	20.12	38.33	<0.001
KFG	27.32	7.83	2.22	2.83	16.82	37.46	<0.001
SOGS	8.68	3.24	0.66	1.00	13.19	35.54	<0.001
BDI	10.48	6.82	5.50	4.33	3.45	50.51	0.001

### Pretest

To construct subjectively meaningful trials for each participant, extensive behavioral testing was done in a pretest session that took place on another day. During pretest, participants performed an adaptive temporal discounting paradigm resulting in one pretest discounting parameter for every participant (Peters and Büchel, 2009, [Bibr B58][Bibr B51][Bibr B50]). Additionally, we interviewed each participant to collect personal relevant, real future events (Peters and Büchel, 2010). Five to seven participant specific future events were selected, with delays ranging from a few days up to ∼200 d. For every event a descriptive label (“episodic tag”) was constructed (e.g., 45 d/“vacation Paris” to refer to a vacation in Paris that the participants has planned 45 d in the future).

### Episodic temporal discounting task

**Figure 1.**
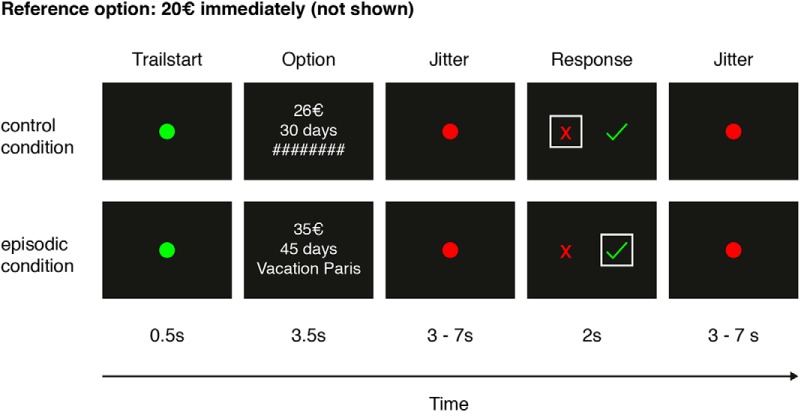
Illustration of one example trial from the control condition (top) and the episodic condition (bottom). Participants made choices between a reference option of 20 euros immediately and LL options. Offers in the control condition consisted of amount and delay, whereas offers in the episodic condition where enriched using participant-specific real future event tags (see Materials and Methods). Selecting check is to accept the LL offer, selecting X is to choose 20 euros immediately. Trials were presented in pseudo-randomized order.

We used a task from a previous study in healthy participants (Peters and Büchel, 2010; Fig. 1), which consisted of two conditions that were randomized trial-wise. The episodic condition involved decisions between a constant immediate reward of 20 euros (not shown) and various delayed rewards (presented on the screen) which were tied to a specific future event, as collected during the pretest session. Trials of the control condition were independent of future events. Control condition delays were drawn from a uniform distribution between one day and the maximum delay of the episodic condition. Episodic and control trials alternated in their time distance. LL reward amounts of half of the trials were chosen to linearly cover the range between 20.5 and 99.5 euros. The other half of LL reward amounts were constructed with respect to the pretest discounting behavior. For each delay, participant specific indifference points were calculated with cognitive modeling (the indifference point specifies the points were SS and LL reward match in subjective value). Amounts of the LL reward were drawn from a normal distribution with the indifference point as mean and a standard deviation of 4.

Before fMRI scanning, participants completed a set of nine practice control trials. Immediately before fMRI, participants were told that episodic tags would be presented during fMRI scanning but no additional instructions regarding the cues were given.

Trials from both conditions were randomized and presented in four sessions during fMRI scanning. After fMRI scanning, participants completed a questionnaire outside the scanner, targeting potential episodic imagination during fMRI scanning. Participants were asked to rate for every event on a six-item Likert scale how often and how vivid they imagined the respective event.

Participants received 10 euros/h cash reimbursement after completion of the fMRI session. Additionally, one trial was randomly selected and paid out in accordance with the participants decision on that trial.

### Cognitive modeling

Choice data were fit with formal models of intertemporal choice, using Bayesian statistics as implemented in the software JAGS 3.4.0 (Plummer, 2003). We used a group level hierarchical estimation scheme. This has been shown to result in parameter estimates that are more reliable with reduced outliers (Ahn et al., 2011; Lee, 2011). We compared hyperbolic discounting (Mazur and Coe, 1987), discounted utility (Pine et al., 2009, 2010), constant sensitivity (Ebert and Prelec, 2007), and intertemporal choice heuristic (Marzilli Ericson et al., 2015) models. Model comparison was done using the Watanabe-Aikine Information Criteria (WAIC; Watanabe, 2010; Vehtari et al., 2015). During MCMC sampling in JAGS, we recorded a log likelihood matrix (trials by samples). This matrix was used to calculate WAIC values with the R package *loo*. Model comparison revealed that hyperbolic discounting (Mazur and Coe, 1987) accounted for the data best (WAIC hyperbolic model: 56,913.42; discounted utility: 1,873,382.76; constant sensitivity: 292,453.95; intertemporal choice heuristic: 115,549.49). This model describes discounting of value over time as a hyperbolic function, with one free parameter *k* to describe the degree of discounting.

This model describes the reduction of subjective value over time as a hyperbola:

$$SV=\frac{A}{1+k*D}$$

Here, *SV* is the subjective (discounted) value of the delayed option, *D* is the delay of the LL reward (in days) and *A* is the reward magnitude of the LL option and *k* is a subject specific discounting parameter. Larger *k* values indicate higher impulsivity. Parameter estimates were used for the statistical analysis of the behavioral data (log-transformed to account for their skewed distribution) and to construct parametrically modulated regressors for the fMRI analysis. To this end, we used each participant’s parameter estimate of *k* to calculate the subjective trial-wise value for presented LL rewards.

Subjective values were transformed into choice probabilities using the softmax action selection:

$$p_{LL}=\frac{exp(SV/\beta )}{exp(20/\beta )+exp(SV/\beta )}$$

This included another free parameter *β* reflecting decision noise (*p_LL_*: probability of choosing the LL option, *SV*: subjective value of the LL option, 20 is the fixed amount of the immediate option).

### fMRI

MRI was collected with a 3T system (Siemens Trio) using a 32-channel head-coil. An average of 1415 volumes per participant were recorded in four sessions and the first five volumes of each session were discarded to allow the BOLD signal to stabilize. Each volume consisted of 40 slices (2 × 2 × 2 mm in-plane resolution and 1-mm gap, repetition time = 2.4 s, echo time = 25 ms). To avoid distortions in the frontal cortex the recorded volumes were flipped by 30° from the anterior and posterior commissures connection line. Participants watched the screen via a head-coil mounted mirror. Additionally, high-resolution T1 weighted structural images were acquired. For one pathological gambler, only three out of four sessions were acquired due to technical problems. For one control participant no structural images were recorded.

MRI data preprocessing and analysis was done using SPM12 (Wellcome Department of Cognitive Neurology, London, United Kingdom). First, all scans of each participant were realigned to the mean volume. Second, to account for the shifted acquisition time of slices within a volume, slice time correction to the onset of the middle slice was performed. Then, all functional images were normalized to Montreal Neurological Institute (MNI) space using affine regularization. Finally, all functional images were smoothed with a Gaussian kernel of 8-mm full-width at half-maximum.

FMRI data were first analyzed using a general linear model (GLM) as implemented in SPM12. On the first level, presentation windows of the LL option were modeled by convolving the duration of presentation (i.e., 3.5 s) with a canonical haemodynamic response function (HRF), separately for control and episodic trials. The subjective value of the LL reward was entered as a first parametric modulator in the first level GLM analysis. As a second parametric modulator, choice LL was entered. Parametric modulators were also convolved with the HRF. Button pressed were modeled separately. We built nuisance regressors using the GLMdenoise toolbox (Kay et al., 2013). GLMdenoise extracts principal components from voxels that are unrelated to the task (i.e., voxels in which the *R* ^2^ is smaller than 0%). The signal in these components is assumed to represent noise (e.g., physiologic noise, movement or scanner artifacts). Principal component scores of the noise components are then included as additional regressors in the GLM to account for task-unrelated variance.

### Dynamic causal modeling (DCM)

Both vmPFC and NAcc have been shown to represent values during decision-making (Haber and Knutson, 2010; Bartra et al., 2013; Clithero and Rangel, 2014). To test the effective connectivity between vmPFC and NAcc during intertemporal choices, we used a DCM approach (Friston et al., 2003). We first extracted the BOLD time course for every participant from both regions of interest (ROIs). The first ROI was defined by the main effect of subjective value in the vmPFC (group conjunction (peak MNI coordinates -4, 56, 0; see Results). The second ROI was defined by the value signal condition difference in the left NAcc (peak MNI coordinates -8, 10, -12; see Results). ROI time courses were extracted within a sphere around the participant specific peak within the ROI (vmPFC 5 mm, NAcc 3 mm in diameter).

To analyze interactions between vmPFC and NAcc, we constructed 16 models (four variations of value input × four variations of episodic modulation), which were grouped into two model-families (Penny et al., 2010): one family including all models with a modulation of the vmPFC to NAcc connection during the episodic condition, and one family including all models without such a modulation. Model families were compared using random effects Bayesian model selection as implemented in SPM12 (Stephan et al., 2009).

## Results

### Sample characteristics and psychopathology

As expected, pathological gamblers differed from healthy controls in all measures of pathological gambling (DSM-5 criteria: *t* = 20.12, df = 38.33, *p* < 0.001, KFG: *t* = 16.82, df = 37.46, *p* < 0.001 and SOGS: *t* = 13.19, df = 35.54, *p* < 0.001). Due to the high positive correlation between KFG and SOGS (*r* = 0.94, *p* < 0.001), we aggregated both measures by averaging z-scores to construct a single pathological gambling score (referred to as “addiction severity”; Wiehler et al., 2015). In addition to gambling addiction, groups differed in depression scores (Beck depression inventory, BDI; Beck et al., 1961), *t* = 3.45, df = 50.51, *p* = 0.001), but not in alcohol and nicotine use (see Table 1 for sample characteristics).

### Behavioral data

**Figure 2.**
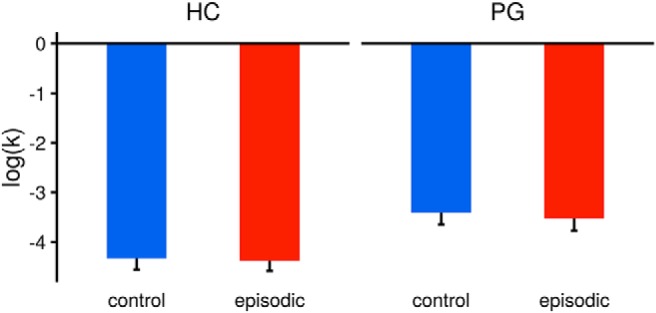
Behavioral results. Estimates of the *k* parameter of the hyperbolic discounting function were obtained for every participant and condition. An ANOVA (see Results) revealed a significant effect of group and a trend-level effect of condition on impulsivity parameters, but no significant group × condition interaction. PG, pathological gamblers; HC, healthy controls. Error bars indicate ±1 standard error of the mean.

For behavioral analyses, we pooled behavioral data of fMRI participants with behavioral data of pilot participants. Participants’ choices were analyzed with cognitive models of temporal discounting and across all participants and conditions, the hyperbolic model (Mazur, 1987) accounted the data best, where larger *k* parameter estimates indicate higher impulsivity. An ANOVA revealed that log(*k*) was different between pathological gamblers and healthy controls (all participants: *F*_(61)_ = 0.7.58, *p* ≤ 0.01, fMRI participants only: *F*_(46)_ = 5.13, *p* = 0.02). Overall, there was a trend-level difference between episodic and control condition (all participants: *F*_(61)_ = 0.3.09, *p* = 0.08, fMRI participants only: *F*_(46)_ = 3.29, *p* = 0.08). The group × condition interaction was not significant (all participants: *F*_(61)_ = 0.61, *p* = 0.44, fMRI participants only: *F*_(46)_ = 0.03, *p* = 0.86; Fig. 2). In the gamblers, log(*k*) parameter estimates from the control condition showed a positive correlation with addiction severity (*r* = 0.34, *p* = 0.03, one-sided). The reduction of impulsivity in the episodic condition (the “tag-effect”; Peters and Büchel, 2010), was quantified as the difference of log(*k*) parameter estimates between conditions. In contrast to previous work (Peters and Büchel, 2010), postscan imagery ratings did not correlate with the tag-effect (pathological gamblers: *r* = 0.17, *p* = 0.35, healthy controls: *r* = -0.05, *p* = 0.79) and did not differ between groups (*t* = -0.87, df = 60.02, *p* = 0.39). 19 out of 31 gamblers and 18 out of 32 controls had a lower *k* parameter estimate in the episodic condition, and these proportions did not differ significantly between groups (χ^2^ = 63, *p* = 1).

**Figure 3.**
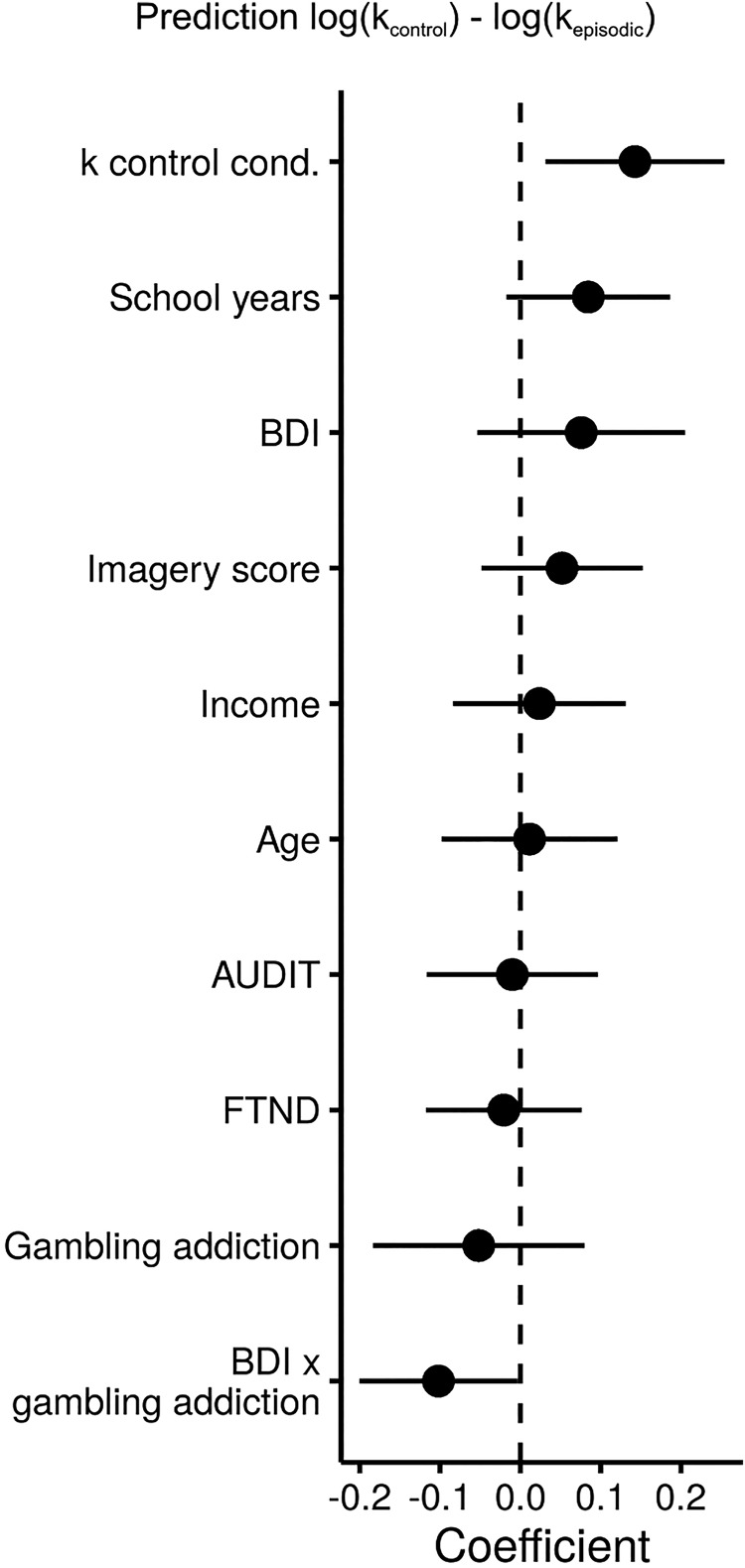
Prediction of log(*k*) differences between experimental conditions. Control condition impulsivity contributed significantly to the prediction of log(*k*) reduction due to episodic tags. The interaction of depression and pathological gambling significantly reduced this tag-effect.

To explore contributions to the variability of the tag-effect, we included the predictors group, age, education (school-years), income, nicotine dependence (FTND), alcohol use [alcohol use disorders identification test (AUDIT)], depression (BDI), post-testing imagery score and control condition log(*k*) parameter in a multiple regression analysis. Among all predictors, baseline discounting (i.e., log(*k*) from the control condition) was predictive of the tag-effect (*b* = 0.14, *p* = 0.01). Depression has been found to affect episodic future thinking and is comorbid in pathological gamblers. Accordingly, the interaction of pathological gambling severity and depression significantly reduced the tag-effect (*b* = -0.10, *p* = 0.04; Fig. 3).

An ANOVA revealed that the softmax *β* parameter, indicating choice stochasticity, was not increased in pathological gamblers (*F*_(61)_ = 0.1.57, *p* = 0.22). Also, *β* parameters were not different in the episodic condition (*F*_(61)_ = 0.0.61, *p* = 0.44) and the group × condition interaction was not significant (*F*_(61)_ = 0.2.28, *p* = 0.14).

### FMRI

#### Brain activations by the episodic condition

**Figure 4.**
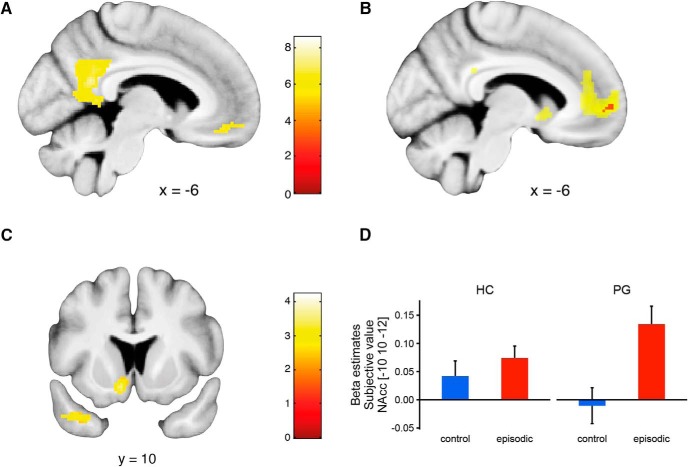
***A***, Categorical analysis (episodic > control condition, conjunction analysis across pathological gamblers and healthy controls, FWE whole-brain peak-level corrected, *p* < 0.05) revealed activity in precuneus and vmPFC. ***B***, Main effect of subjective value pooled across both experimental conditions, conjunction across pathological gamblers and healthy controls. Red, FWE whole-brain peak-level corrected, *p* < 0.05; yellow, *p* < 0.001 uncorrected. ***C***, Increased value signals in the episodic condition compared with the control condition in the left NAcc, *p* < 0.01 uncorrected for display purposes. ***D***, β estimates extracted from the peak voxel of ***C*** in the left NAcc (this plot serves to visualize the direction of effects, not for statistical inference). PG, pathological gamblers; HC, healthy controls. Error bars indicate ±1 standard error of the mean.

In both gamblers and controls, an extensive midline-network showed increased activation for the episodic versus control trials (*p* < 0.05 FWE corrected at the peak level for whole-brain volume, conjunction across groups; Nichols et al., 2005; Fig. 4*A*). Specifically, we observed activations in brain regions previously implicated in episodic future thinking (Schacter et al., 2007; Peters and Büchel, 2010), such as retrosplenial cortex/PCC (peak MNI coordinates -2, -54, 20, z = 6.49) and vmPFC (peak MNI coordinates -6, 42, -14, z = 5.44). At a lower threshold (*p* < 0.001 uncorrected) additional activations in middle temporal gyrus, angular gyrus and operculum were visible; while the lack of multiple comparison correction means that we do not know the false positive risk of the latter activations, we report them for comparisons for future studies with higher statistical power.

#### Representations of subjective value

Model-based estimates of subjective values of the LL options were included as a parametric modulator in the fMRI analysis to test for a correlation of BOLD signal with subjective reward values. We replicated a previously reported main effect of subjective value (Peters and Büchel, 2009; Bartra et al., 2013; Clithero and Rangel, 2014), pooled across conditions in the vmPFC (*p* < 0.05 FWE corrected at the peak level, conjunction across groups, peak MNI coordinates -4, 56, 0, z = 5.44). At a lower threshold (*p* < 0.001 uncorrected) additional activations were visible in the NAcc (peak MNI coordinates -8, 8, -6, z = 4.35), and PCC (peak MNI coordinates -2, -42, 26, z = 4.01; Fig. 4*B*).

Previous research suggested both task-dependent enhancements and attenuations in valuation responses in gamblers (Miedl et al., 2012; Leyton and Vezina, 2013), focusing on the ventral striatum/NAcc. Here, we did not observe any overall group differences. However, focusing on the NAcc, we identified voxels showing a stronger activation with subjective values in the episodic condition compared with the control condition in both groups (peak MNI coordinates -10, 10, -12, z = 3.21, p = 0.023, peak-level corrected for multiple comparisons within the accumbens area neuromorphometrics mask as implemented in SPM12; Fig. 4*C*). A *post hoc* test revealed that this increase of valuation signals in the episodic condition was driven by the pathological gamblers (peak MNI coordinates -8, 12, -10, z = 3.27, p_SVC_ = 0.010, same mask; Fig. 4*D*) while healthy controls provide no significant supra-threshold clusters for the same contrast in the NAcc.

**Figure 5.**
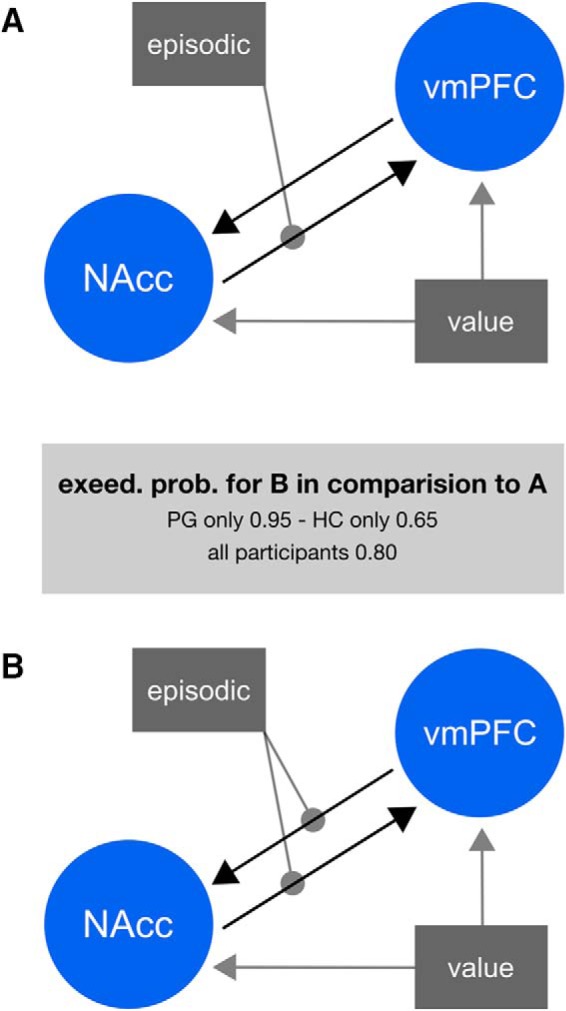
Model families to describe vmPFC and NAcc interactions. All models grouped in family ***A*** assume no modulation of the vmPFC to NAcc connection by the episodic condition. All models in family ***B*** assume such a modulation. Gray arrows denote connections, modulations or driving inputs that may or may not exist. Exceed. prob., exceedance probability (see Results for more details). PG, pathological gamblers; HC, healthy controls.

A DCM analysis allowed us to further explore this condition by value interaction in pathological gamblers. Given the main effect of subjective value in vmPFC, an increased value signal in the ventral striatum/NAcc during the presentation of episodic tags and strong anatomic connections between both regions (Haber and Knutson, 2010), we hypothesized that the connection between vmPFC and NAcc is modulated by the episodic condition. We created models with all possible combinations of value inputs and modulations and grouped them the into two model families (Penny et al., 2010): one family including all models assuming a modulation of the vmPFC to NAcc connection by the episodic condition and one family including all models assuming no such modulation (Fig. 5). Bayesian model selection revealed an exceedance probability (exp) of 0.952 for the first family in pathological gamblers (the exceedance probability of a model family denotes the probability that, conditional on the available data and the chosen model family space, this family has a higher posterior probability than any other model family considered). The same pattern reoccurred for the healthy controls (exp = 0.648) and across all participants (exp = 0.8053). Taken together, this analysis provides evidence that the episodic condition modulates valuation related brain connectivity in pathological gamblers.

**Figure 6.**
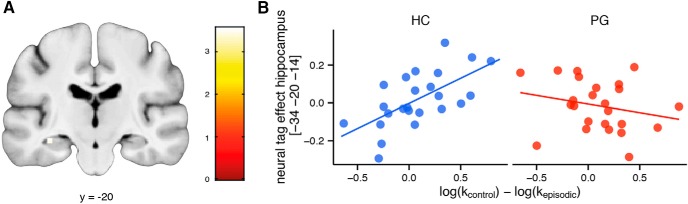
***A***, Cluster in the left hippocampus that codes a conjunction of (1) a correlation between the neural tag-effect (subjective value during episodic > control) and the behavioral tag-effect (log(*k*) control – log(*k*) episodic) in healthy participants; and (2) that this correlation is larger in healthy participants compared with patients. ***B***, Scatter-plot illustrating tag-effect correlations in the left hippocampus separate for healthy controls (HC) and pathological gamblers (PG).

Previous research found an interaction effect reflected by activity in the hippocampus, with a modulation effect of prospection on valuation (Peters and Büchel, 2010; Benoit et al., 2011). We thus investigated a correlation of the neuronal tag-effect (subjective value in the episodic > control condition) with the size of the behavioral tag-effect (log(*k*) control - log(*k*) episodic) as in previous work (Peters and Büchel, 2010). A cluster in the left hippocampus showed such a correlation only in healthy controls, a significant positive correlation in healthy controls in conjunction with this correlation being significantly larger in controls than in gamblers (-34, -20, -14, z = 3.31, p_SVC_ = 0.044, peak-level corrected for multiple comparisons within the Neuromorphometrics anatomic mask of the left hippocampus; Fig. 6).

## Discussion

An attenuating effect of episodic future thinking on temporal discounting has been found in numerous studies (Peters and Büchel, 2010; Benoit et al., 2011; Cheng et al., 2012; Daniel et al., 2013a, 2013b; Lin and Epstein, 2014; Palombo et al., 2015; Sasse et al., 2015). To the best of our knowledge, this is the first study to explore this effect and the underlying neuronal mechanisms in pathological gamblers. Episodic tags were shown along with LL rewards in a temporal discounting task. Tags activated a similar prospection related brain network in pathological gamblers and healthy controls and tended to attenuate discounting behavior likewise. We found that striatal valuation signals increase during the presentation of episodic tags in pathological gamblers, an effect driven by a functional modulation of the vmPFC to NAcc connection. Behavioral changes due to episodic tags were correlated with valuation signal changes in the hippocampus in healthy controls, but not pathological gamblers.

In healthy participants, an induction of episodic future thinking attenuates temporal discounting (Peters and Büchel, 2010; Benoit et al., 2011; Cheng et al., 2012; Daniel et al., 2015; Palombo et al., 2015). In our study, this effect was only trend-level significant across all participants (*p* = 0.08) with a small effect size of *d* = 0.33 in pathological gamblers and *d* = 0.12 in matched healthy controls compared with a medium effect size of 0.4 in a previous sample of healthy young adults tested with the same task (Peters and Büchel, 2010). Both pathological gamblers and healthy controls showed a similar variability in the effect. Healthy controls in the present study differed from a student population (such as the one tested in the original paper; Peters and Büchel, 2010). Controls were matched to the pathological gamblers on age, income, education, nicotine use. Lower education might influence the vividness of imaginations. Higher incomes in our study might change the utility of the rewards presented in our study compared with studies in healthy young students. Finally, in contrast to the original paper, the present study included only male participants. These factors might likely contribute to the increased variability in the tag-effect in the present sample.

During the presentation of episodic tags about personal future events, both pathological gamblers and controls showed robust activations in PCC/precuneus and vmPFC and reported spontaneous imaginations, as in a previous study using the same task (Peters and Büchel, 2010). This network has been implicated in episodic prospection and construction processes (Schacter et al., 2012). In line with previous behavioral research (Wiehler et al., 2015), but distinct from findings in long-term opiate users (Mercuri et al., 2015), we found no evidence for an impairment of episodic future thinking in pathological gamblers and no evidence for an impairment in the underlying neuronal mechanisms.

Exploratory multiple regression revealed two predictors for the tag-effect in our study. First, participants with high discounting in the control condition showed a larger reduction of impulsivity in the episodic condition. This could point toward an increased effect of the episodic condition in high impulsivity participants, independent of a pathological gambling diagnosis. Nevertheless, a regression to the mean effect could also contribute to the observed reduction of high impulsivity. Second, interacting with addiction severity, depressive symptoms reduced the tag-effect. Major depression disorder, as comorbid in some gamblers tested here, can affect episodic future thinking (King et al., 2011; Hach et al., 2014) and is associated with attenuated activation of the default mode network during episodic future thinking (Hach et al., 2014). Our experimental manipulation might thus be affected by comorbid depression in pathological gambling. Investigating the tag-effect in major depression patients could shed more light on the role of depression in episodic future thinking during decision-making.

The role of striatal reward and valuation signals in pathological gambling is still debated (Clark et al., 2013). Both diminished (Comings and Blum, 2000; Reuter et al., 2005; Balodis et al., 2012b) and enhanced (Hewig et al., 2010; Van Holst et al., 2012) striatal responses in pathological gambling have been reported. Given the role that contextual factors can play in modulating neural value signals (Leyton and Vezina, 2012; Miedl et al., 2014; Paliwal et al., 2014), it is possible that such factors account for these differences. We observed neuronal value representations across both groups in PCC and vmPFC (Peters and Büchel, 2009; Bartra et al., 2013; Clithero and Rangel, 2014). As analysis of neural value signals depend on regressors constructed through cognitive modeling, it is possible that group differences were over-estimated in previous studies. Furthermore, an attenuation of value signals with increasing pathological gambling severity was reported previously (Reuter et al., 2005; Miedl et al., 2012), but was not observed in our study. However, the previously reported effects have been largely driven by a few highly addicted gamblers (Reuter et al., 2005; Miedl et al., 2012), which were not part of the present data set (although all gamblers in our study fulfilled the DSM-5 criteria of pathological gambling).

Across all subjects, we observed increased value signals in the left ventral striatum in the episodic condition and a *post hoc* test revealed that this effect was driven by pathological gamblers. This cluster is close to findings of a previous study, which reported diminished striatal valuation signals in gamblers and found a negative correlation with impulsivity (Balodis et al., 2012a). This is in line with other previous research that found reduced valuation signals for nongambling cues (similarly to our study, as all episodic tags were strictly nongambling) and enhanced valuation signals for gambling cues (Leyton and Vezina, 2013). To our best knowledge, we provide first results showing a manipulation to enhance valuation signals in pathological gamblers in a nongambling context. These findings are of high clinical interest, as reduced valuation signals for nongambling rewards have been identified as a hall mark feature of gambling addiction and reliable interventions to restore striatal valuation signals are missing (Leyton and Vezina, 2013).

We identified the hippocampus as a region to link changes of neuronal valuation signal with the size of the behavioral tag-effect (Peters and Büchel, 2010; Lebreton et al., 2013). The hippocampus has been identified to contribute to both episodic future thinking and decision-making and might be an important node linking between these processes (Cheung and Cardinal, 2005; Johnson et al., 2007; Mariano et al., 2009). Our findings suggest, that this link might be affected in pathological gambling, as the correlation between hippocampal activation and the size of the behavioral tag-effect was significantly larger in the control group than in the patients (in the controls, it was also significantly larger than zero). Future studies might investigate the role of the hippocampus in impulsive decision-making in pathological gambling in greater detail.

Taken together, we investigated for the first time a modulation of temporal discounting by episodic future thinking in a group of nontreatment seeking pathological gamblers. pathological gamblers were overall more impulsive than healthy controls, but neuronal mechanisms of episodic future thinking were surprisingly similar between groups. We observed intact valuation signals in the vmPFC of pathological gamblers. The functional connection from vmPFC to NAcc was modulated by the episodic condition, resulting in enhanced striatal valuation signals in pathological gamblers. By fostering episodic future thinking during decision-making about nongambling options, it might be possible to increase the valuation of nongambling options and rewards in pathological gamblers.

## Synthesis

Reviewing Editor: Karen Szumlinski, University of California at Santa Barbara Decisions are customarily a result of the Reviewing Editor and the peer reviewers coming together and discussing their recommendations until a consensus is reached. When revisions are invited, a fact-based synthesis statement explaining their decision and outlining what is needed to prepare a revision will be listed below. Note: If this manuscript was transferred from JNeurosci and a decision was made to accept the manuscript without peer review, a brief statement to this effect will instead be what is listed below.

After careful editorial review of the revised manuscript, I deem this report acceptable for publication in eNeuro. The authors made a strong effort to address all of the concerns raised by Reviewer 2 and in my opinion, have satisfied all of the concerns. I did note however, a minor typo at the bottom of the Abstract that can be corrected during the proof stage: please correct "We provide first evidence..." to "We provide the first evidence..." . Congratulations on the acceptance of your report.
